# The boundaries between complex posttraumatic stress disorder symptom clusters and post-migration living difficulties in traumatised Afghan refugees: a network analysis

**DOI:** 10.1186/s13031-022-00455-z

**Published:** 2022-04-27

**Authors:** Jennifer Schiess-Jokanovic, Matthias Knefel, Viktoria Kantor, Dina Weindl, Ingo Schäfer, Brigitte Lueger-Schuster

**Affiliations:** 1 Department of Clinical and Health Psychology, Faculty of Psychology, University of Vienna, Wächtergasse 1, 1010 Vienna, Austria; 2 Department of Psychiatry and Psychotherapy, University Medical Centre Hamburg-Eppendorf, Hamburg, Germany

**Keywords:** Complex posttraumatic stress disorder (CPTSD), Refugees, Post-migration stressors, Emotion regulation, Language acquisition, Re-experiencing, Integration, Network analysis

## Abstract

**Background:**

Psychological distress due to the ongoing war, violence, and persecution is particularly common among Afghan asylum seekers and refugees. In addition, individuals face a variety of post-migration living difficulties (PMLDs). Complex posttraumatic stress symptoms are among the most common mental health problems in this population, and were associated with the overall burden of PMLDs. The complex interplay of posttraumatic symptoms has been investigated from a network perspective in previous studies. However, individuals are embedded in and constantly react to the environment, which makes it important to include external factors in network models to better understand the etiology and maintaining factors of posttraumatic mental health problems. PMLDs are a major risk factor for posttraumatic distress and considering their impact in interventions might improve response rates. However, the interaction of these external factors with posttraumatic psychopathological distress is not yet fully understood. Thus, we aimed to illuminate the complex interaction between PMLDs and CPTSD symptom clusters.

**Objective:**

The main objective is the exploration of the network structure and the complex interplay of ICD-11 CPTSD symptom clusters and distinct forms of PMLDs.

**Method:**

The symptom clusters of CPTSD and PMLDs were collected within a randomised controlled trial among 93 treatment-seeking Afghan asylum seekers and refugees via a fully structured face-to-face and interpreter assisted interview. Using a network analytical approach, we explored the complex associations and network centrality of the CPTSD symptom clusters and the PMLD factors: discrimination & socio-economical living conditions, language acquisition & barriers, family concerns, and residence insecurity.

**Results:**

The results suggest direct links within and between the constructs (CPTSD, PMLD). Almost all PMLD factors were interrelated and associated to CPTSD, family concerns was the only isolated variable. The CPTSD symptom cluster re-experiencing and the PMLD factor language acquisition & barriers connected the two constructs. Affective dysregulation had the highest and avoidance the lowest centrality.

**Conclusions:**

Re-experiencing and affective dysregulation have the strongest ties to PMLDs. Thus, these domains might explain the strong association of posttraumatic psychopathology with PLMDs and, consequently, prioritization of these domains in treatment approaches might both facilitate treatment response and reduce burden caused by PMLDs.

**Supplementary Information:**

The online version contains supplementary material available at 10.1186/s13031-022-00455-z.

## Introduction

The long-awaited serenity after arrival in the host country in Europe can often not be achieved, as many Asylum seekers and refugees (AS&R) are confronted with the physical and mental consequences of exposures to the ongoing war, human rights violations, and persecution [1–3]. Compared to AS&R from other countries of origin, individuals from Afghanistan in Austria reported particularly poor health trajectories over time. The authors suggested that these findings might reflect marginalization processes of subgroups of AS&R and an interdependence of origin- and country-specific conditions [4], such as a lower level of education, longer waiting periods for positive asylum decisions [5], and a higher rejection rate of asylum applications compared to Syrian refugees [6]. In addition to the challenge of dealing with health problems and potentially traumatic experiences [7], those affected have to adapt to a new environment and face various postmigration living difficulties (PMLDs) [8–10].

Drožđek [11] postulated the integrative contextual model for AS&R, which assumes that an individual is embedded in multiple, interconnecting dimensions in which individuals operate and constantly respond to their environment. Risk factors, such as the broad construct of PMLDs are considered as dynamic system localised on each of these dimensions: intraindividual (e.g. loneliness, homesickness), interpersonal (e.g. conflicts, family separation), societal (e.g. discrimination, housing conditions), and cultural (e.g. language, asylum law; [11]). The development and psychopathological expression of mental health problems is assumed to be bidirectionally related to these dimensions and might change over time [7, 11, 12].

Along with depression and anxiety disorders, post-traumatic stress disorder (PTSD) is one of the most commonly reported mental disorders among refugees in high-income countries [13, 14]. Prevalence rates, while highly heterogeneous, are significantly higher than in the non-refugee population or among people living in conflict and war zones [14]. In ICD-11, the PTSD diagnosis is more narrowly defined compared to DSM-5 and comprises three symptom clusters (re-experiencing, avoidance, sense of current threat). Additionally, ICD-11 introduced a twin diagnosis, complex post-traumatic stress disorder (CPTSD), which is intended to better encompass the sequelae of complex traumatic experiences and includes all PTSD symptom clusters and additionally three symptom clusters related to disturbances of self-organization (DSO; affect dysregulation, negative self-concept, difficulties in interpersonal relationships). The diagnoses must be associated with functional impairments in various domains of life [15]. Treatment-seeking AS&R samples report higher prevalence rates of CPTSD (16–38%) than the general refugee population [16].

Overall, the negative effect of PMLDs on mental health and quality of life was found to be at least equal to or even exceeding that of potentially traumatic experiences [9, 17]. Accordingly, higher distress due to PMLDs overall [18], but also due to individual PMLDs such as uncertain visa status [19], was associated with higher levels of complex posttraumatic stress disorder (CPTSD). Deeper investigations of this relationship point out that while potentially traumatic experiences were related with posttraumatic stress disorder (PTSD), total distress due to PMLDs showed an relationship with disturbances of self-organisation (DSO) [20]. An analysis of the associations of various PMLD factors with CPTSD suggested that while *language acquisition & barriers* predicted the membership to the CPTSD subgroup, the other PMLD factors were equally present in both investigated subgroups [21]. These findings suggest individual boundaries between the symptom clusters of CPTSD and different forms of PMLDs.

In recent years, the ongoing debate about the ontology of psychopathology and whether mental disorders are caused by a common cause (latent variable/common cause model) or rather by the interaction of individual symptoms (network approach) has led to the rejection of both extreme versions and the adoption of hybrid models [22]. Consistent with the integrative contextual model for AS&R [11], the hybrid models link both models and assume that potentially traumatic experiences or stressors act as a common cause that triggers multiple CPTSD and PTSD symptoms, which in turn may interact with each other and lead to additional symptoms until a self-perpetuating network emerges [22–24]. So-called feedback loops are already known from clinical observations. A stimulus and the related interpretation and response, ultimately influence the further development of the original stimulus (X > Y > Z > X). An example would be a bodily sensation which, when misinterpreted, leads to increased anxiety and ultimately to an increased bodily sensation [23]. Such feedback loops in networks may involve not only intrapersonal processes but also interact with unique sociocultural factors and context that affect understanding, attribution, and response to potentially traumatic experiences and PMLDs [11, 25, 26]. As a consequence, resources and the ability of adaptive coping might be reduced [27].

While several studies have already examined the complex interplay of the individual symptoms of PTSD [28] and CPTSD [29–32], and generalisability has been examined in various samples with different characteristics and cultural backgrounds [29, 30], there is only one initial study investigating how individual PMLDs are related to each other [32]. Overall, there is a lack of knowledge on how external variables influence network structure and dynamics in psychopathology [23, 32, 33].

Only a few studies have considered the interaction between distinct symptom domains and external stressors when examining mental health problems in refugees [34–36]. De Schryver et al. [35] reported that although symptoms and potentially traumatic experiences/stressors formed different clusters in the network, they were directly connected. Overall, traumatic experiences, daily stressors, basic needs, safety [35, 36], and social problems [34] showed the highest centrality. Regarding symptoms, sleep, hopelessness, melancholy, and nightmares were central [36]. To our knowledge, there is no study investigating the interaction between PMLDs and CPTSD symptom clusters in AS&R so far.

The known association between CPTSD and PMLDs in general [18] provides insufficient insight into the underlying dynamics. The practice-oriented question of which types of PMLDs are associated with which CPTSD symptom clusters is so far unresolved. This study aimed to promote a better understanding of these dynamics and provide an initial contribution to the selection of intervention targets in order to improve AS&R health care. Therefore, the aim of this study was to explore the boundaries of the CPTSD symptom clusters with the external PMLD factors, and investigate their centrality indices in a cross-sectional sample of traumatised Afghan AS&R living in Austria. We presumed connections within and between the CPTSD and PMLD constructs.

## Methods

### Participants

We used data from baseline assessments of a randomised controlled trial evaluating a psychological intervention (PIAAS Study; [37]) conducted between July 2019 and December 2020. The total sample included 93 adult, treatment-seeking AS&R from different areas of Afghanistan with Dari as their first language. Exclusion criteria were screening with an emotional distress score below the cut-off value (RHS-15 score < 12 or distress thermometer < 5), ongoing trauma-focused treatment, significant cognitive impairment, and current mental or physical conditions requiring other treatment and/or impeding participation (e.g. acute suicidality, psychotic symptoms). The ethics committee of the University of Vienna ethically approved the study (Reference Numbers: 00356 and 00445).

### Measures

The fully structured interview was conducted with a trained psychologist and an interpreter. German and Dari versions of the International Trauma Questionnaire (ITQ) and the Post-migration living difficulties Checklist (PMLDC) were administered. Likert scales were additionally presented visually for better understanding and to support illiterate participants.

### CPTSD symptoms

The six symptom clusters of CPTSD, comprising the three symptom clusters of PTSD (re-experiencing, avoidance, sense of current threat) and the three symptom clusters of the disturbances in self-organisation (DSO; affective dysregulation, negative self-concept, disturbances in relationships), were surveyed in relation to the level of symptom distress experienced in the past month using the ITQ. Participants answered two items per cluster, twelve items in total, on a five-point Likert scale (0 = "*Not at all*" to 4 = "*Extremely*"). Higher scores indicate higher levels of burden. The ITQ has good psychometric properties [38]. The Cronbach's alpha coefficient in this study was 0.89.

### Post-migration living difficulties

The multitude of different PMLDs AS&R face in the host country were assessed with the support of the PMLD checklist [8]. We adapted and extended the already used Dari/German PMLDC version [20] to the Austrian context, finally containing a total of 26 items (see Additional file 1: Table S1). Additional items were translated and back-translated by trained interpreters following the golden standard of translation guidelines [39]. The frequency of experiencing the specific PMLDs in the past month was recorded on a five-point Likert scale (0 = "*Not at all*" to 4 = "*Extremely*"). The Cronbach's alpha coefficient in this study was 0.77.

### Analysis

**Fig. 1:**
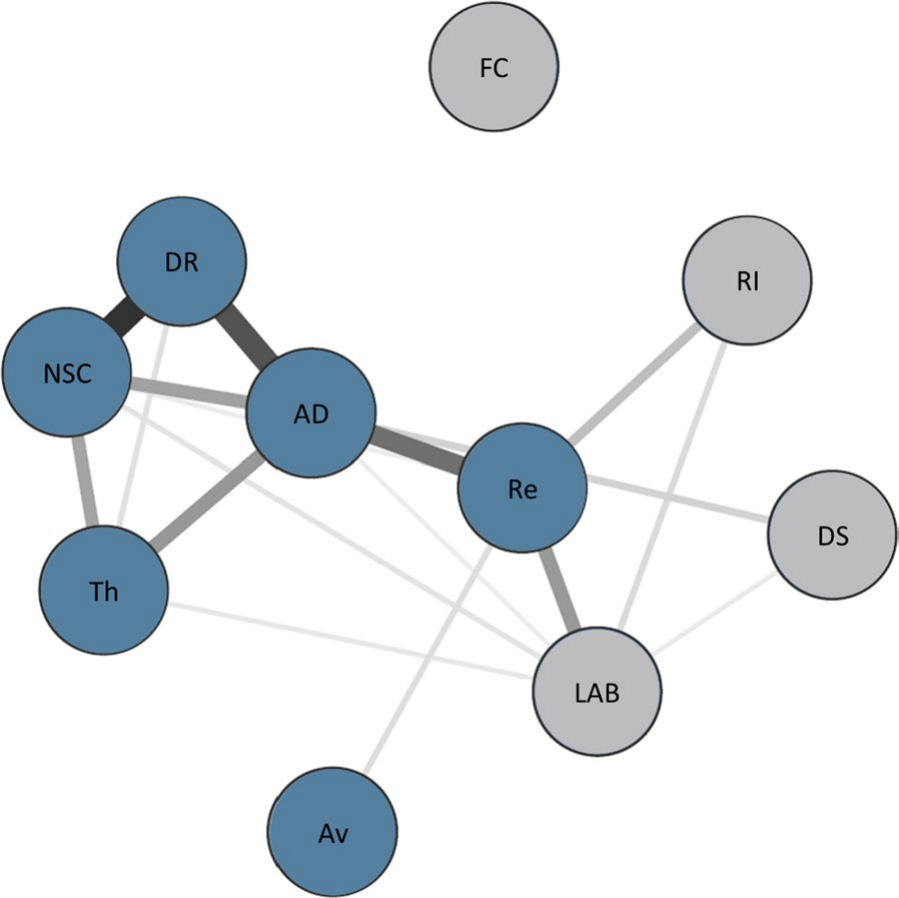
Network estimation. *Note:* Regularized partial correlation network of the CPTSD symptom cluster and distinct PMLDs. Edge thickness represents the strength of association. All edges indicate positive relationships. Re = “Re-experiencing”, Av = “Avoidance”, Th = “Sense of current Threat”, AD = “Affective Dysregulation”, NSC = “Negative Self-Concept”, DR = “Disturbances in relationships”, DS = “Discrimination & Socio-economical living conditions”, LAB = “Language Acquisition & Barriers”, FC = “Family Concerns”, RI = “Residence Insecurity”

The network structure of the six CPTSD symptom clusters and the four PMLD factors was estimated (Fig. 1). To reduce the number of items, data-driven PMLD factors were calculated in a previous study ([15], see Additional file 1: Figure S1 for item assignment) using a regularised exploratory factor analysis developed specifically for small samples [40]. The network was estimated with the R package qgraph [41] and the robustness and bridge centrality of the network were examined using the R packages *bootnet* and *networktools* [42]. All analyses were performed using R (version 4.1.0; [43]).

### Network estimation

The presented network was estimated using a Gaussian graphical model [44]. Partial correlations between the total of ten nodes (re-experiencing (Re), avoidance (Av), sense of current threat (Th), affective dysregulation (AD), negative self-concept (NSC), disturbances in relationships (DR), socio-economical living conditions & discrimination (SC), language acquisition & barriers (LAB), family concerns (FC), residence insecurity (RI)) are represented with edges. The line thickness of the edges represent the strength of the association. In a first step, a correlation matrix based on polychoric correlations was calculated ([45]; see Additional file 1: Table S2). In a second step, the network was estimated using EBICglasso, an estimation method to minimise false positive detection of connections. To visualise the estimated network, the Fruchterman-Reingold algorithm [46] was used in the third step, which places nodes with more and/or stronger connections closer together. The higher the degree of a centrality (strength, predictability), the conceptually closer a node is to all other nodes in a network [47]. In addition bridge centrality (bridge strength, bridge predictability) were calculated. These parameters estimate the strength of each node in connecting the constructs CPTSD and PMLDs.

### Network stability

Following the recommended three steps [45], the R package bootnet was used to estimate the stability of the network. Firstly, we calculated 95% confidence intervals using 1000 bootstrap iterations to test edge certainty and significance between edge weights. Secondly, to estimate the stability of the order of centrality indices, we used a node-dropping sub setting bootstrap technique and the correlation stability (CS-) coefficient, which is an index of the stability of centrality indices. Values above 0.25 indicate sufficient stability. In a final step, we calculated the bootstrapped edge-weight and centrality difference test for the networks to check whether these differ significantly from each other.

### Missing data

For the CPTSD data, a total of 3.3% (3.2–4.3%) individual data points were missing stemming from four incomplete cases. A total of 10 incomplete cases resulted in 3.6% (3.2–5.4%) missing values in the PMLDC data. Three cases with more than 50% missing data were completely deleted. To deal with all other missing values, an algorithm from the R package "mice" was used, which estimates values by single imputation (predictive mean matching) using Fully Conditional Specifications [48].

## Results

### Descriptive statistics

Data from 93 treatment-seeking Afghan AS&R were included in the network analysis. The mean age was 34.77 years (SD = 13.84), with 45.2% female participants. About half of the participants were married or living in a partnership (51.7%). A high illiteracy rate was found, with over 60% of participants having no formal education (37.6%) or having attended only elementary school (22.6%). The overall employment rate was 26.8%, the majority reported no employment (61.3%), including 22.6% due to lack of a work permit. On average, participants had been in Austria for 5.9 years (SD = 4.36) and 52.7% reported uncertain asylum status. There were 12.07 (SD = 5.03) potentially traumatic experience types reported on average. 50% of the participants met criteria for CPTSD, while 17.78% individuals met criteria for PTSD only (see Additional file 1: Table S3 for further sociodemographic characteristics and the frequency of potentially traumatic experience types). The average scores in each symptom cluster and PMLD can be found in Table 1.

**Table 1:** Means and standard deviations of the CPTSD symptom clusters & PMLDs

Variables	M (SD)
Re-experiencing (Re)	5.03 (2.15)
Avoidance (Av)	4.63 (2.03)
Sense of current threat (Th)	4.78 (2.05)
Affective dysregulation (AD)	4.63 (1.89)
Negative self-concept (NSC)	4.20 (2.54)
Disturbances in relationships (DR)	3.99 (2.24)
Discrimination & socio-economical living conditions (DS)	10.62 (4.00)
Language acquisition & barriers (LAB)	16.14 (4.05)
Family concerns (FC)	13.32 (3.99)
Residence insecurity (RI)	9.83 (3.69)

M, mean; SD, standard deviation

### Network estimation

**Fig. 2:**
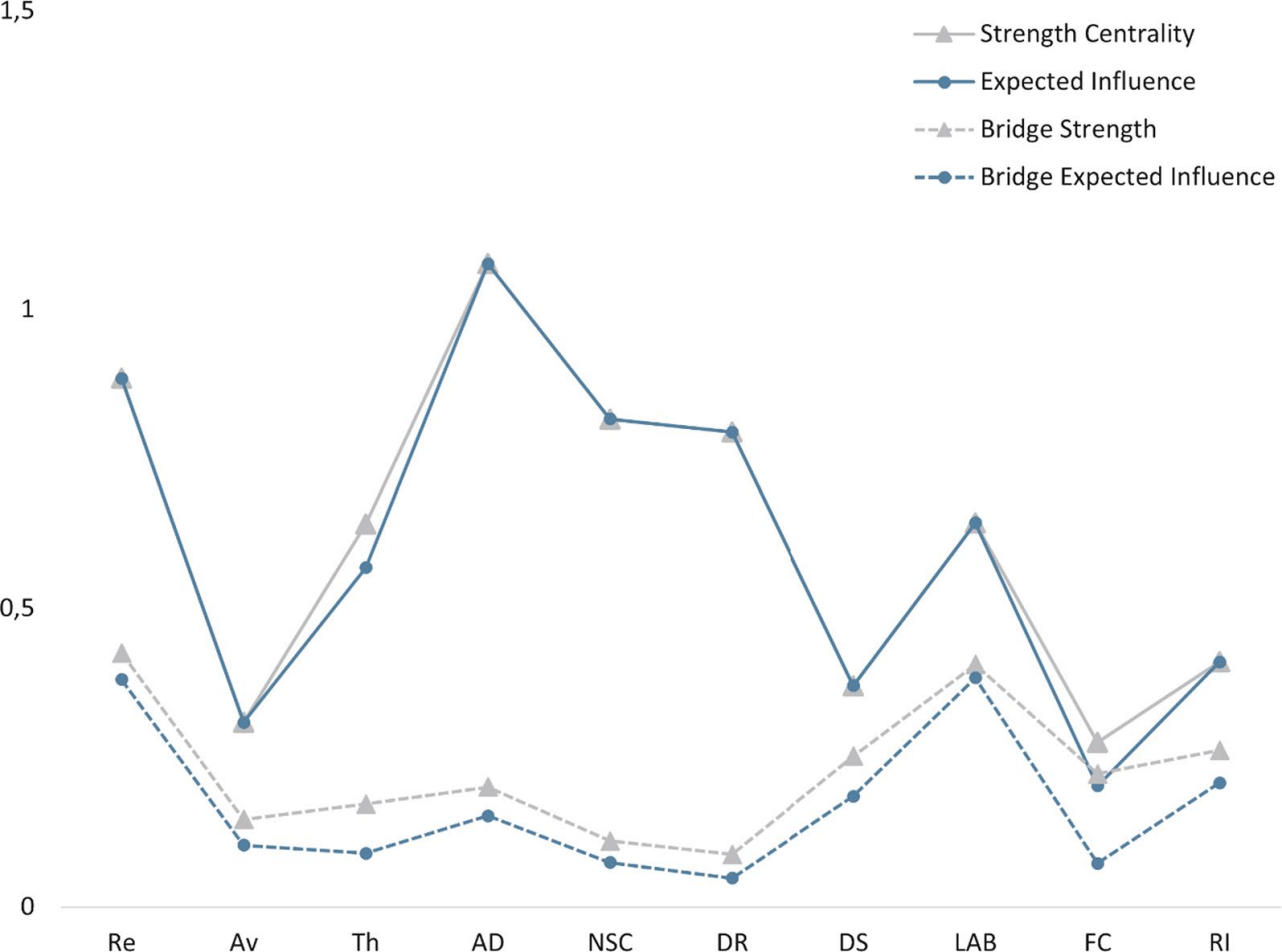
Centrality indices. *Note:* Centrality and bridge centrality measures for each variable were estimated by standardised node strength and predictability. For definitions of abbreviations, see Fig. 1

The structure of the network of the six symptom clusters of CPTSD and the four PMLD factors is shown in Fig. 1. 17 of 45 possible edges were estimated above zero. All associations were positive and there was only one isolated node (FC) in the network, i.e. all other nodes were directly or indirectly connected via other nodes. Three of the PMLD factors were interconnected: PMLD factor LAB was associated with DS and RI. LAB and RI showed the strongest connection to Re, other weaker connections could be found between LAB and NSC, DR and Th. DS was connected to AD. While the three symptom clusters of DSO were connected to each other, the three symptom clusters of PTSD were not interconnected. Re and Av were connected, Th was only connected to all DSO clusters. Since the strength centrality measure had the highest reliability and were closely related to the predictability, the interpretation was based on this centrality measure. Overall, the three nodes with the highest strength centrality were the three CPTSD clusters: AD, Re, and NSC. The connection between negative self-concept and disturbances in relationships was the strongest within the entire network. The nodes with the highest bridge expected influence and bridge strength were Re and LAB, which means that Re was the CPTSD symptom cluster with the strongest average connection to the PMLDs, and LAB was the PMLD factor with the strongest average connection to CPTSD. The centrality and bridge centrality estimates for all nodes are presented in Fig. 2.

### Network stability

The network showed large bootstrap confidence intervals around the edge weights, which partly overlapped (see Additional file 1: Figure S2), indicating that their order should be interpreted with caution. The CS-coefficient (CS-Coeff _(cor = 0.7)_ = 0.44) for the strength centrality metric exceeded the recommended minimum cut-off of 0.25 in the network suggesting a moderate stability [45]. In addition, we checked whether the edges or the node strength differed significantly (see Additional file 1: Figures S3, S4). The results showed that many of the nodes did not differ significantly in strength.

## Discussion

To our knowledge, this is the first study investigating the boundaries between CPTSD symptom clusters and different forms of PMLDs in a highly distressed sample of treatment-seeking Afghan AS&R. In line with our hypothesis, the results clearly illustrate that there is an association between psychopathology and external factors, that differ among the various forms of PMLDs. Overall, we found that the PMLD factors *discrimination & socio-economical life conditions*, *language acquisition & barriers*, and *residence insecurity* were associated with each other and with individual CPTSD symptom clusters. *Family concerns* was isolated in the network and showed no associations. A further finding was that while all DSO clusters were interrelated, for PTSD symptom clusters, *avoidance* showed a relationship with *re-experiencing*, but *sense of current threat* was exclusively related to DSO symptom clusters. The strongest linkages between the CPTSD and PMLD constructs, seem to be the two PMLD factors *language acquisition & barriers* as well as *residence insecurity* and the CPTSD cluster *re-experiencing*. The latter was directly linked to the PMLD factors and mediated their relationship to each other and *affective dysregulation*. Which, as the most central CPTSD factor was also directly related to *discrimination & socioeconomical life conditions*.

Consistent with the integrative contextual model for AS&R [11] and previous investigations examining the associations between PMLDs and psychopathology [35, 36], almost all PMLD factors were connected to CPTSD symptom clusters. Although associations were found between PMLDs and DSO symptom clusters, in contrast to the findings of Hecker et al. [20], the strongest associations were found to a PTSD cluster.

One of the most compelling findings was the association between the CPTSD symptom cluster *re-experiencing* and the PMLD factor *language acquisition & barriers*. These two domains showed the strongest associations between the CPTSD and PMLD constructs. This finding confirms initial findings that demonstrated an association between the PTSD symptom cluster re-experiencing and language [49]. Intrusive re-experiencing, including trauma-associated flashbacks or nightmares, represents a core symptom of CPTSD and PTSD, which in investigations of PTSD symptom networks, has been repeatedly identified as particularly central [28].

Successful adaptation in the host country with participation in social life and access to various facilities is closely related to proficiency in the local language. The factor *language acquisition & barriers* included distress and dependence on others in various situations requiring language, such as health service utilization or official channels. This factor also revealed a link to *current sense of threat, negative self-concept, affective dysregulation, discrimination & socio-economical life conditions,* and *residence insecurity*, latter included higher stress due to insecure residence status as well as greater fear of future deportation. The persistent feeling of insecurity [36], especially in the context of insecure residence associated with PTSD [19] has been reported several times and might hinder the calming and processing of potentially traumatic experiences.

Afghanistan has one of the highest illiteracy rates worldwide, numerous individuals do not have access to educational opportunities in their home country. Almost 60% of our participants had no formal education or had only completed elementary school. Those Afghan AS&R in Austria might be confronted with Western educational institutions and concepts of learning for the first time and face the challenge of having to start literacy right away with a foreign language.

Another aspect is that language acquisition is a complex process involving different cognitive abilities related to encoding and retrieval. Successful encoding depends on various executive skills (e.g., attention, working memory; [50]) [51]. Consistent with our findings, Lansing et al. [52] reported a positive association between re-experiencing and increased difficulties with verbal learning. In line with the found mediation effect of *re-experiencing* between *language acquisition & barriers* and *affective dysregulation*, deficits in cognitive resources lead to difficulties in calming down and thus reducing intrusions [52].

Anxiety, helplessness, and feelings of loss of control are common reactions associated with potentially traumatic experiences. These emotions may accompany many interpersonal situations in everyday life or during the asylum process in which language comprehension or the ability to express oneself as well as actively respond to the environment through language is limited. And, as previously reported, might be associated with an increased risk of discrimination [32]. We hypothesise that these mentioned feelings, combined with a reduced ability to respond actively, and reinforced by *affective dysregulation*, may trigger *re-experiencing.*

In addition to the important link between *re-experiencing* and *affective dysregulation*, links to various CPTSD clusters and *language acquisition & barriers* to *negative self-concept* have been found. A negative self-concept and reduced self- efficacy were found to be associated with potentially traumatic experiences [53]. The exposure of a variety of situations associated with language barriers or difficulties in language acquisition might further negatively impact self-concept, which in turn might promote avoidance of such situations, and further impeding language acquisition and social inclusion.

Affective dysregulation has been repeatedly reported as a key factor in traumatised AS&R [54, 55] and has been identified as a mediator in the relationship between potentially traumatic experiences and PTSD as well as PMLDs [54]. Consistent with these findings, as already mentioned *affective dysregulation* showed the highest centrality in our study and was associated directly with almost all CPTSD symptom clusters and the PMLD factor *discrimination and socioeconomic living conditions*.

Discrimination, as well as problems related to finances or housing, jeopardise human needs and might be accompanied by vast emotional distress. Furthermore, Santangelo et al. [56] found that people with PTSD in particular have an affective baseline with higher distress and less positive emotionality in comparison to healthy individuals. Affective variability indicated a more intense response to internal or external but less to positive processes or events and it took longer for regulatory or homeostatic processes to restore deviant affective fluctuations to baseline and allow emotional recovery. Following these results, we hypothesised that individuals with higher levels of affective dysregulation might respond stronger to existential concerns in addition to normal responses [56, 57]. Conversely, emotional outbursts and emotional numbing could further increase the risk for such stressful experiences. Lack of emotional clarity, difficulties with goal-directed behaviour, rumination and suppression may play a particularly important role [54, 55, 58, 59] and may also be a link to further psychopathology.

Following *affective dysregulation, re-experiencing, and negative self-concept* were symptom clusters with high strength centrality, and presented with the strongest connection within the network. This was in line with the findings of CPTSD networks [29]. Negative self-concept, especially feelings of worthlessness have an immense impact on the development, persistence and unfavourable prognosis of PTSD [60, 61]. There might be an interaction between these areas such as that a *negative self-concept* increases the risk of social withdrawal and in turn a lack of positive relational experiences reinforces *the negative self-concept*. Overall, refugees have a higher risk of loneliness and low social support, factors that have been repeatedly related to higher levels of mental distress [62], CPTSD [63] and lower quality of life [64]. Additionally, we suggest that numbness due to *affective dysregulation* and an associated reduced self-awareness or ability to engage in interpersonal relationships might further promote *disturbances in relationships* and a *negative self-concept*.

Interestingly, the PMLD factor *family concerns* was isolated in the network. Coming from a culture where social structures such as the family are of particular importance, longing for the family and the country of origin as well as worries about loved ones appear independent of CPTSD symptomatology in the estimated network. However, these reactions might be related to other psychopathologies, quality of family relationships or demographic variables, such as marital status or family separation. This is particularly interesting as different forms of PMLDs might be associated to varying degrees with mental disorders such as CPTSD and some PMLDs might be stressors independent of any psychopathology associated with normal situationally adequate distress of the challenging life situation.

### Strengths and limitations

The major strength of the study lies in the examination of one of the most burdened, hard-to-reach population of treatment-seeking AS&R in Austria. Interpreter-assisted and face-to-face fully structured interviews conducted by a trained clinical psychologist reduced language barriers and psychological distress during the assessments.

Nevertheless, some limitations need to be considered. The results must be interpreted with caution, as the sample was relatively small and generalizability to other ethnic groups or regional differences cannot be guaranteed. Problems in understanding the meaning of particular symptoms might bias the results and could potentially explain some associations such as between re-experiencing and language barriers. The cross-sectional study design does not allow conclusions about directionality. We conclude, consistent with the integrative contextual model for AS&R [11], that it is highly likely that central symptoms are reciprocally related to their neighbours. The question of whether interventions should address central symptoms to increase treatment success and achieve improved symptom relief has recently been debated [23, 24]. To date, there is no agreement; an important research goal would be to address this issue through intervention studies with longitudinal within-person networks. Until then, central symptoms should be considered as potentially important treatment targets when considering personalised diagnosis and intervention [23, 24, 29].

The Covid-19 pandemic interrupted the assessment process and might have influenced symptom severity. Therefore, future replications of the study with larger samples would be required, here the application of Latent Network Modelling (LNM) could contribute to an improved control of measurement errors [45]. In addition, inclusion of further psychopathologies, such as depression and anxiety, and a longitudinal design in refugee samples with different ethnic background are recommended to further investigate the generalizability and directionality of the results, thus relationships might change over time [7, 11, 12].

### Clinical implications

The main findings of this study underline the crucial importance of including PMLDs and its complex interplay with CPTSD into AS&R assessment and treatment strategies [65]. Psychosocial interventions have already been shown to be effective in several studies [66]. An individual assessment of the burden due to specific PMLDs (e.g. *language acquisition & barriers, discrimination & socioeconomic living conditions*) and a according focus on these and their individual interaction with CPTSD symptoms, such as *affective dysregulation* or *re-experiencing*, might represent important treatment targets. Reducing the burden of PMLDs and improving symptom management in daily life might reduce the burden caused by PMLDs and CPTSD. Furthermore, interventions that promote social connectedness, such as community-based interventions or group therapy, might be additionally beneficial. A more precise assessment of trauma-associated re-experiencing, affective dysregulation, negative self-concept, and disturbances in relationships should be integrated into the psychological assessments and subsequently into the treatment strategies of traumatised AS&Rs. A combination of trauma-focused and non-trauma-focused treatment approaches might be beneficial [67]. Especially a focus on the reduction of trauma-associated nightmares and intrusive memories as well as to promote the ability to cope with the symptoms and to stay present might be advantageous [68]. So far, there are few treatment recommendations addressing re-experiencing directly in CPTSD [69]. Establishing trauma-sensitive language courses and integrating interventions to promote cognitive receptivity and reduce stress could be another interdisciplinary task for educators and psychologists [49].

## Conclusion

Our results provide initial evidence for various associations that might be important for a better understanding of the overall relationship between PMLDs and CPTSD. Psychological treatment to improve mental health, quality of life, functioning as well as adaptation in the host country appear to be particularly important. Due to the localisation of PMLDs at different levels, such as the cultural/governmental level, promoting trauma-sensitive language courses or policies to reduce discrimination or tedious asylum procedures might have a positive impact on mental and physical health.

## Supplementary Information

**Additional file 1:** Post-Migration Living Difficulties Checklist (PMLDC), Correlation Matrix, Sociodemographic characteristics, Item assignment to PMLD factors, Edge Accuracy Analysis, Edge weights difference test, Centrality Stability Analysis, Centrality difference Test.

## Data Availability

The data that supports the findings of this study are openly available in “Zenodo” at https://doi.org/10.5281/zenodo.5054032.

## Abbreviations

AS&R: Asylum seekers and refugees
CPTSD: Complex posttraumatic stress disorder
DSO: Disturbances in self-organisation
Re: Re-experiencing
Av: Avoidance
Th: Current sense of threat
AD: Affective dysregulation
NSC: Negative self-concept
DR: Disturbances in relationships
PMLDs: Post-migration living difficulties
DS: Discrimination & socio-economical living conditions
LAB: Language acquisition & barriers
FC: Family concerns
RI: Residence insecurity
PMLDC: Post-Migration Living Difficulties Checklist
ITQ: International Trauma Questionnaire

## References

[1] Alemi Q, James S, Cruz R, Zepeda V, Racadio M (2014). Psychological distress in Afghan refugees: a mixed-method systematic review. J Immigr Minor Health.

[2] Bogic M, Njoku A, Priebe S (2015). Long-term mental health of war-refugees: a systematic literature review. BMC Int Health Hum Rights.

[3] Scoglio AAJ, Salhi C. Violence exposure and mental health among resettled refugees: a systematic review. Trauma Violence Abuse. 2020:1524838020915584. 10.1177/1524838020915584 32238052

[4] Georges D, Buber-Ennser I, Rengs B, Kohlenberger J, Doblhammer G (2021). Health determinants among refugees in Austria and Germany: a propensity-matched comparative study for Syrian, Afghan, and Iraqi refugees. PLoS ONE.

[5] Eggenhofer-Rehart PM, Latzke M, Pernkopf K, Zellhofer D, Mayrhofer W, Steyrer J (2018). Refugees' career capital welcome? Afghan and Syrian refugee job seekers in Austria. J Vocat Behav.

[6] BMI. Asylstatistik: Bundesministerium für Inneres; 2020 [cited 2021 Sep 16]. https://www.bmi.gv.at/301/Statistiken/files/Jahresstatistiken/Asyl_Jahresstatistik_2020.pdf.

[7] Blanchard MA, Heeren A. Ongoing and future challenges of the network approach to psychopathology: from theoretical conjectures to clinical translations. In: Reference module in neuroscience and biobehavioral psychology. Elsevier; 2020.

[8] Silove D, Sinnerbrink I, Field A, Manicavasagar V, Steel Z (1997). Anxiety, depression and PTSD in asylum-seekers: assocations with pre-migration trauma and post-migration stressors. Br J Psychiatry.

[9] Li SSY, Liddell BJ, Nickerson A (2016). The Relationship Between Post-Migration Stress and Psychological Disorders in Refugees and Asylum Seekers. Curr Psychiatry Rep.

[10] Schweitzer R, Melville F, Steel Z, Lacherez P (2006). Trauma, post-migration living difficulties, and social support as predictor of psychological adjustment in resettled Sudanese refugees. Aust NZ J Psychiatry.

[11] Drožđek B (2015). Challenges in treatment of posttraumatic stress disorder in refugees: towards integration of evidence-based treatments with contextual and culture-sensitive perspectives. Eur J Psychotraumatol.

[12] Neal JW, Neal ZP. Nested or networked? Future directions for ecological systems theory. Soc Dev. 2013:n/a–n/a.

[13] Turrini G, Purgato M, Ballette F, Nosè M, Ostuzzi G, Barbui C (2017). Common mental disorders in asylum seekers and refugees: Umbrella review of prevalence and intervention studies. Int J Ment Health Syst.

[14] Henkelmann J-R, de Best S, Deckers C, Jensen K, Shahab M, Elzinga B (2020). Anxiety, depression and post-traumatic stress disorder in refugees resettling in high-income countries: systematic review and meta-analysis. BJPsych Open.

[15] World Health Organization. The 11th Revision of the International Classification of Diseases: World Health Organization; 2019 [cited March 3rd, 2017]. https://icd.who.int/browse11/l-m/en.

[16] de Silva U, Glover N, Katona C (2021). Prevalence of complex post-traumatic stress disorder in refugees and asylum seekers: systematic review. BJPsych Open.

[17] Hynie M (2018). The social determinants of refugee mental health in the post-migration context: a critical review. Can J Psychiatry.

[18] Tay AK, Mohsin M, Rees S, Tam N, Kareth M, Silove D (2018). Factor structures of complex posttraumatic stress disorder and PTSD in a community sample of refugees from West Papua. Compr Psychiat.

[19] Liddell BJ, Nickerson A, Felmingham KL, Malhi GS, Cheung J, Den M et al. Complex posttraumatic stress disorder symptom profiles in traumatized refugees. J Trauma Stress. 2019. 10.1002/jts.22453 31648412

[20] Hecker T, Huber S, Maier T, Maercker A (2018). Differential associations among PTSD and complex PTSD symptoms and traumatic experiences and postmigration difficulties in a culturally diverse refugee sample. J Trauma Stress.

[21] Schiess-Jokanovic J, Knefel M, Kantor V, Weindl D, Schäfer I, Lueger-Schuster B (2021). Complex post-traumatic stress disorder and post-migration living difficulties in traumatised refugees and asylum seekers: the role of language acquisition and barriers. Eur J Psychotraumatol.

[22] Fried EI, Cramer AOJ (2017). Moving forward: challenges and directions for psychopathological network theory and methodology. Perspect Psychol Sci.

[23] McNally RJ (2021). Network analysis of psychopathology: controversies and challenges. Annu Rev Clin Psychol.

[24] Fried EI, Eidhof MB, Palic S, Costantini G, Huisman-van Dijk HM, Bockting CLH (2018). Replicability and generalizability of Posttraumatic Stress Disorder (PTSD) networks: a cross-cultural multisite study of PTSD symptoms in four trauma patient samples. Clin Psychol Sci.

[25] Yuval K, Aizik-Reebs A, Lurie I, Demoz D, Bernstein A. A functional network perspective on posttraumatic stress in refugees: implications for theory, classification, assessment, and intervention. Transcult Psychiatry. 2020:1363461520965436. 10.1177/1363461520965436 33292082

[26] Miller KE, Rasmussen A (2017). The mental health of civilians displaced by armed conflict: an ecological model of refugee distress. Epidemiol Psychiatr Sci.

[27] Hou WK, Liu H, Liang L, Ho J, Kim H, Seong E (2019). Everyday life experiences and mental health among conflict-affected forced migrants: a meta-analysis. J Affect Disord.

[28] Birkeland MS, Greene T, Spiller TR (2020). The network approach to posttraumatic stress disorder: a systematic review. Eur J Psychotraumatol.

[29] Knefel M, Karatzias T, Ben-Ezra M, Cloitre M, Lueger-Schuster B, Maercker A (2019). The replicability of ICD-11 complex post-traumatic stress disorder symptom networks in adults. Br J Psychiatry.

[30] Knefel M, Lueger-Schuster B, Bisson JI, Karatzias T, Kazlauskas E, Roberts NP (2020). A cross-cultural comparison of icd-11 complex posttraumatic stress disorder symptom networks in Austria, the United Kingdom, and Lithuania. J Trauma Stress.

[31] Knefel M, Tran US, Lueger-Schuster B (2016). The association of posttraumatic stress disorder, complex posttraumatic stress disorder, and borderline personality disorder from a network analytical perspective. J Anxiety Disord.

[32] Wicki B, Spiller TR, Schick M, Schnyder U, Bryant RA, Nickerson A (2021). A network analysis of postmigration living difficulties in refugees and asylum seekers. Eur J Psychotraumatol.

[33] Cramer AOJ, van Borkulo CD, Giltay EJ, van der Maas HLJ, Kendler KS, Scheffer M (2016). Major depression as a complex dynamic system. PLoS ONE.

[34] Jayawickreme N, Mootoo C, Fountain C, Rasmussen A, Jayawickreme E, Bertuccio RF (2017). Post-conflict struggles as networks of problems: a network analysis of trauma, daily stressors and psychological distress among Sri Lankan war survivors. Soc Sci Med.

[35] de Schryver M, Vindevogel S, Rasmussen AE, Cramer AOJ (2015). Unpacking constructs: a network approach for studying war exposure, daily stressors and post-traumatic stress disorder. Front Psychol.

[36] Mootoo C, Fountain C, Rasmussen A (2019). Formative psychosocial evaluation using dynamic networks: trauma, stressors, and distress among Darfur refugees living in Chad. Confl Heal.

[37] Knefel M, Kantor V, Schiess-Jokanovic J, Weindl D, Schäfer I, Lueger-Schuster B (2020). A brief transdiagnostic psychological intervention for Afghan asylum seekers and refugees in Austria: a randomized controlled trial. Trials.

[38] Cloitre M, Shevlin M, Brewin CR, Bisson JI, Roberts NP, Maercker A (2018). The international trauma questionnaire: development of a self-report measure of ICD-11 PTSD and complex PTSD. Acta Psychiatr Scand.

[39] Bontempo R (1993). Translation fidelity of psychological scales: an item response theory analysis of an individualism-collectivism scale. J Cross Cult Psychol.

[40] Jung S, Lee S (2011). Exploratory factor analysis for small samples. Behav Res Methods.

[41] Epskamp S, Cramer AOJ, Waldorp LJ, Schmittmann VD, Borsboom D. qgraph: network visualizations of relationships in psychometric data. J Stat Softw 2012; 48(4).

[42] Epskamp S. Package ‘bootnet’: Bootstrap Methods for Various Network Estimation Routines; 2015. https://cran.r-project.org/web/packages/bootnet/index.html.

[43] R Core Team. R: A Language and Environment for Statistical Computing. Vienna, Austria: R Foundation for Statistical Computing; 2020. http://www.R-project.org/.

[44] Lauritzen SL (1996). Graphical models, Oxford statistical science series.

[45] Epskamp S, Kruis J, Marsman M (2017). Estimating psychopathological networks: be careful what you wish for. PLoS ONE.

[46] Fruchterman TMJ, Reingold EM (1991). Graph drawing by force-directed placement. Softw Practice Exp.

[47] Opsahl T, Agneessens F, Skvoretz J (2010). Node centrality in weighted networks: generalizing degree and shortest paths. Soc Netw.

[48] van Buuren S, Groothuis-Oudshoorn K. mice: multivariate imputation by chained equations in R. J Stat Softw. 2011;45(3).

[49] Kartal D, Alkemade N, Kiropoulos L (2019). Trauma and mental health in resettled refugees: mediating effect of host language acquisition on posttraumatic stress disorder, depressive and anxiety symptoms. Transcult Psychiatry.

[50] Gathercole SE, Baddeley AD (2014). Working Memory and Language Processing.

[51] Samuelson KW (2011). Post-traumatic stress disorder and declarative memory functioning: a review. Dialogues Clin Neurosci.

[52] Lansing AE, Plante WY, Golshan S, Fenemma-Notestine C, Thuret S (2019). Emotion regulation mediates the relationship between verbal learning and internalizing, trauma-related and externalizing symptoms among early-onset, persistently delinquent adolescents. Learn Individ Differ.

[53] Weindl D, Knefel M, Glück TM, Tran US, Lueger-Schuster B (2018). Motivational capacities after prolonged interpersonal childhood trauma in institutional settings in a sample of Austrian adult survivors. Child Abuse Negl.

[54] Nickerson A, Bryant RA, Schnyder U, Schick M, Mueller J, Morina N (2015). Emotion dysregulation mediates the relationship between trauma exposure, post-migration living difficulties and psychological outcomes in traumatized refugees. J Affect Disord.

[55] Koch T, Ehring T, Liedl A (2020). Effectiveness of a transdiagnostic group intervention to enhance emotion regulation in young Afghan refugees: a pilot randomized controlled study. Behav Res Ther.

[56] Santangelo PS, Limberger MF, Stiglmayr C, Houben M, Coosemans J, Verleysen G (2016). Analyzing subcomponents of affective dysregulation in borderline personality disorder in comparison to other clinical groups using multiple e-diary datasets. Borderline Personal Disord Emot Dysregul.

[57] Graham JR, Calloway A, Roemer L (2015). The buffering effects of emotion regulation in the relationship between experiences of racism and anxiety in a Black American Sample. Cogn Ther Res.

[58] Karatzias T, Shevlin M, Hyland P, Brewin CR, Cloitre M, Bradley A (2018). The role of negative cognitions, emotion regulation strategies, and attachment style in complex post-traumatic stress disorder: implications for new and existing therapies. Br J Clin Psychol.

[59] Doolan EL, Bryant RA, Liddell BJ, Nickerson A (2017). The conceptualization of emotion regulation difficulties, and its association with posttraumatic stress symptoms in traumatized refugees. J Anxiety Disord.

[60] Bryant RA, Guthrie RM (2007). Maladaptive self-appraisals before trauma exposure predict posttraumatic stress disorder. J Consult Clin Psychol.

[61] Dunmore E, Clark DM, Ehlers A (2001). A prospective investigation of the role of cognitive factors in persistent Posttraumatic Stress Disorder (PTSD) after physical or sexual assault. Behav Res Ther.

[62] Wang J, Mann F, Lloyd-Evans B, Ma R, Johnson S (2018). Associations between loneliness and perceived social support and outcomes of mental health problems: a systematic review. BMC Psychiatry.

[63] Fox R, Hyland P, Coogan AN, Cloitre M, McHugh PJ (2022). Posttraumatic stress disorder, complex PTSD and subtypes of loneliness among older adults. J Clin Psychol.

[64] Belau MH, Becher H, Kraemer A (2021). Loneliness as a mediator of social relationships and health-related quality of life among refugees living in North Rhine-Westphalia, Germany. BMC Public Health.

[65] Minihan S, Liddell BJ, Byrow Y, Bryant RA, Nickerson A (2018). Patterns and predictors of posttraumatic stress disorder in refugees: a latent class analysis. J Affect Disord.

[66] Turrini G, Purgato M, Acarturk C, Anttila M, Au T, Ballette F et al. Efficacy and acceptability of psychosocial interventions in asylum seekers and refugees: systematic review and meta-analysis. Epidemiol Psychiatr Sci. 2019:1–13. 10.1017/S2045796019000027 PMC6669989 30739625

[67] Schäfer I, Gast U, Hofmann A, Knaevelsrud C, Lampe A, Liebermann P (2019). S3-Leitlinie Posttraumatische Belastungsstörung.

[68] Brewin CR, Gregory JD, Lipton M, Burgess N (2010). Intrusive images in psychological disorders: characteristics, neural mechanisms, and treatment implications. Psychol Rev.

[69] Iyadurai L, Visser RM, Lau-Zhu A, Porcheret K, Horsch A, Holmes EA (2019). Intrusive memories of trauma: a target for research bridging cognitive science and its clinical application. Clin Psychol Rev.

