# Supplementary material for: The boundaries between complex posttraumatic stress disorder symptom clusters and post-migration living difficulties in traumatised Afghan refugees: a network analysis
Source: Confl Health. 2022 Apr 27;16:19. doi: 10.1186/s13031-022-00455-z (PMC9043511; doi:10.1186/s13031-022-00455-z)
Supplement: Supplementary file 1 — Additional file 1: Post-Migration Living Difficulties Checklist (PMLDC), Correlation Matrix, Sociodemographic characteristics, Item assignment to PMLD factors, Edge Accuracy Analysis, Edge weights difference test, Centrality Stability Analysis, Centrality difference Test. [file 13031_2022_455_MOESM1_ESM.docx]

# Supplementary Material:

The boundaries between complex posttraumatic stress disorder and post-migration living difficulties in traumatised Afghan refugees: a network analysis

Jennifer Schiess-Jokanovic^*a^, Matthias Knefel^a^, Viktoria Kantor^a^, Dina Weindl^a^,

Ingo Schäfer^b^, and Brigitte Lueger-Schuster^a^

Author Note

^a^ Department of Clinical and Health Psychology, Faculty of Psychology, University of Vienna, Vienna, Austria.

^b^ Department of Psychiatry and Psychotherapy, University Medical Centre Hamburg-Eppendorf, Hamburg, Germany.

*Correspondence concerning this article should be addressed to Jennifer Schiess-Jokanovic, Department of Clinical and Health Psychology, Faculty of Psychology, University of Vienna, Wächtergasse 1, 1010 Vienna, Austria. E-Mail: [jennifer.schiess-jokanovic@univie.ac.at](mailto:jennifer.schiess-jokanovic@univie.ac.at)

Table S1

*Post-Migration Living Difficulties Checklist (PMLDC)*

| **Items** |
| --- |
| 1. Communication difficulties |
| 1. Discrimination |
| 1. Conflicts with own or other ethnic groups in Austria |
| 1. Family separation |
| 1. Concern for family members remaining in the home country or living far away |
| 1. Impossibility to travel home in case of emergency |
| 1. Difficulties with work (e.g.: work permit, working conditions) |
| 1. Difficulties with official channels (e.g.: interview with asylum agency) |
| 1. Conflicts with authorities |
| 1. No recognition as a refugee |
| 1. Fear of future deportation to the homeland |
| 1. Worries about not receiving medical support or treatment for health problems |
| 1. Not having enough money to buy food, necessary clothing, or pay rent |
| 1. Difficulties in obtaining financial support |
| 1. Loneliness, boredom or isolation |
| 1. Difficulties learning German |
| 1. Difficulties in obtaining adequate housing |
| 1. Family pressure, expectations which cannot be fulfilled ^a^ |
| 1. No contact with family and friends in the country of origin ^a^ |
| 1. Stressful media reports and social media content ^a^ |
| 1. Negative media reports about Afghan fellow citizens in Austria ^a^ |
| 1. Dependence on others due to language (loss of autonomy) ^a^ |
| 1. Ethnic stigmatization ^a^ |
| 1. Difficulties in understanding bureaucratic processes in Austria ^a^ |
| 1. Different social norms than in the country of origin ^a^ |
| 1. Homesickness ^a^ |

*Note.* The post-migration living difficulties checklist was adapted for the study. ^a^ additional item.

Table S2

*Correlation Matrix*

| **Variable** | **1** | **2** | **3** | **4** | **5** | **6** | **7** | **8** | **9** | **10** |
| --- | --- | --- | --- | --- | --- | --- | --- | --- | --- | --- |
| **1. Re** | ­ |  |  |  |  |  |  |  |  |  |
| **2. Av** | .258 | ­ |  |  |  |  |  |  |  |  |
| **3. Th** | .182 | .188 | ­ |  |  |  |  |  |  |  |
| **4. AD** | .467 | .196 | .441 | ­ |  |  |  |  |  |  |
| **5. NSC** | .320 | .078 | .425 | .506 | ­ |  |  |  |  |  |
| **6. DR** | .269 | .091 | .376 | .570 | .599 | ­ |  |  |  |  |
| **7. DS** | .0778 | .172 | .206 | .285 | .125 | .108 | ­ |  |  |  |
| **8. LAB** | .387 | .142 | .271 | .304 | .288 | .168 | .245 | ­ |  |  |
| **9. FC** | .201 | .139 | -.084 | -.030 | -.027 | .033 | .135 | .083 | ­ |  |
| **10. RI** | .317 | .004 | .130 | .229 | .131 | .217 | .119 | .279 | .160 | ­ |

*Note.* Polychoric correlations were used

Table S3

*Sociodemographic characteristics*

| **Sample Characteristics** | **n (%)** |
| --- | --- |
| Female | 42 (45.2 |
| **Educational Level** |  |
| No formal education | 35 (37.6) |
| Elementary school | 21 (22.6) |
| Secondary school | 16 (17.2) |
| High school | 16 (17.2) |
| University | 4 (4.3) |
| **Marital status** |  |
| Single | 37 (39.8) |
| Married/Cohabiting | 48 (51.7) |
| Divorced/widowed | 7 (7.6) |
| **Employment** |  |
| Employed or in training | 7 (7.5) |
| Unemployed/ Unemployed without work permit | 57 (61.3) |
| Student | 11 (11.8) |
| Unable to work/ permanently ill | 2 (2.2) |
| Retired | 2 (2.2) |
| Unpaid housework, childcare | 2 (2.2) |
| Other (e.g., community service) | 7 (7.5) |
| **Asylum Status** |  |
| Granted asylum | 40 (43.0) |
| Subsidiary protection | 18 (19.4) |
| Asylum seeker/  asylum seeker appealing rejection | 27 (29.0) |
| Austrian citizenship | 3 (3.2) |
| Other | 4 (4.3) |
| **Presumptive diagnosis** |  |
| Presumptive ICD-11 CPTSD diagnosis | 45 (50) |
| Presumptive ICD-11 PTSD diagnosis | 16 (17.78) |
|  | **Mean (SD)** |
| Age | 34.77 (13.84) |
| Potentially traumatic experience types | 12.07 (SD=5.03) |
| **Potentially traumatic experience type** | **n (%)** |
| Lack of food or water | 46 (51.69) |
| Ill health without access to medical care | 43 (48.31) |
| Life-threatening disease | 31 (34.83) |
| Lack of shelter | 40 (44.94) |
| Serious accident (for example industrial, agricultural or traffic accident) | 40 (44.94) |
| Fire or explosion | 47 (52.81) |
| Exposure to toxic substances (for example, hazardous chemical radiation) | 11 (12.36) |
| Imprisonment | 27 (30.34) |
| Serious injury | 39 (43.82) |
| Combat situation | 50 (56.18) |
| Staying in a war zone as a civilian | 63 (70.79) |
| Brain washing | 9 (10.11) |
| Violent attack by a family member or a known person (e.g.: assaulted, physically attacked, shot or threatened with a weapon). | 42 (47.19) |
| Violent attack by an unknown person  (e.g.: assaulted, physically attacked, shot or threatened with a weapon). | 50 (56.18) |
| Rape or sexual abuse | 12 (13.48) |
| Other unwanted or undesired sexual experience | 20 (22.47) |
| Natural disaster (e.g.: flood, hurricane, tornado or strong earthquake) | 42 (47.19) |
| Stay in a refugee camp | 59 (66.29) |
| Being exposed to danger during flight (sea, boat, border) | 76 (85.39) |
| Forced isolation from others | 30 (33.71) |
| Being close to death | 49 (55.06) |
| Forced separation of family members | 35 (39.33) |
| Murder of a family member or friend | 54 (60.67) |
| Unnatural death of family members or friends | 50 (56.18) |
| Murder of one or more unknown persons | 45 (50.56) |
| Disappear or be abducted | 25 (28.09) |
| Torture | 36 (40.45) |
| Serious injury, damage or death you have caused to someone else | 7 (7.87) |
| Another situation that was very scary or where you felt your life was in danger (e.g. domestic violence) | 45 (50.56) |

*Note. N = 93.* Numbers may not sum to total N and percentages may not sum to 100 due to missing values.

Figure S1

*Item assignment to PMLD factors*


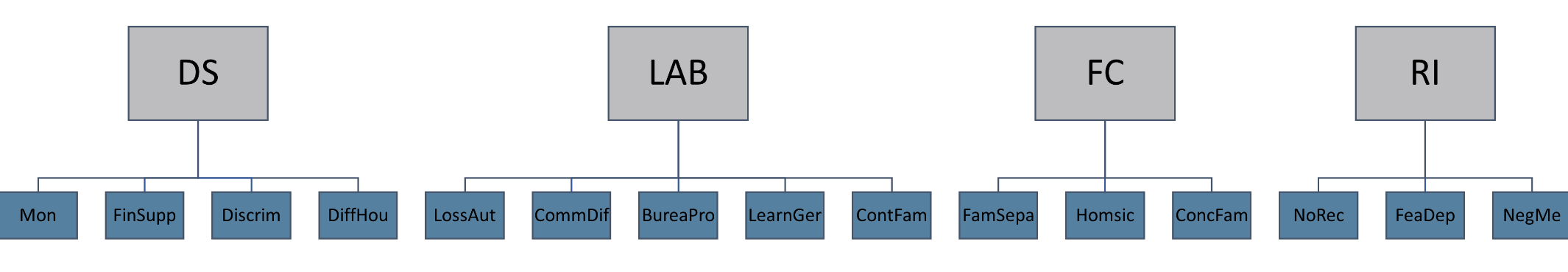


*Note.* Mon = Not having enough money to buy food, necessary clothing, or pay rent, FinSupp = Difficulties in obtaining financial support, Discrim = Discrimination, DiffHou = Difficulties in obtaining adequate housing, LossAut = Dependence on others due to language (loss of autonomy) ^a^, CommDif = Communication difficulties, BureaPro = Difficulties in understanding bureaucratic processes in Austria ^a^, LearnGer = Difficulties learning German, ContFam = No contact with family and friends in the country of origin ^a^ , FamSepa = Family separation, Homsic = Homesickness ^a^, ConcFam = Concern for family members remaining in the home country or living far away, NoRec = No recognition as a refugee, FeaDep = Fear of future deportation to the homeland, NegMe = Negative media reports about Afghan fellow citizens in Austria ^a^.

*
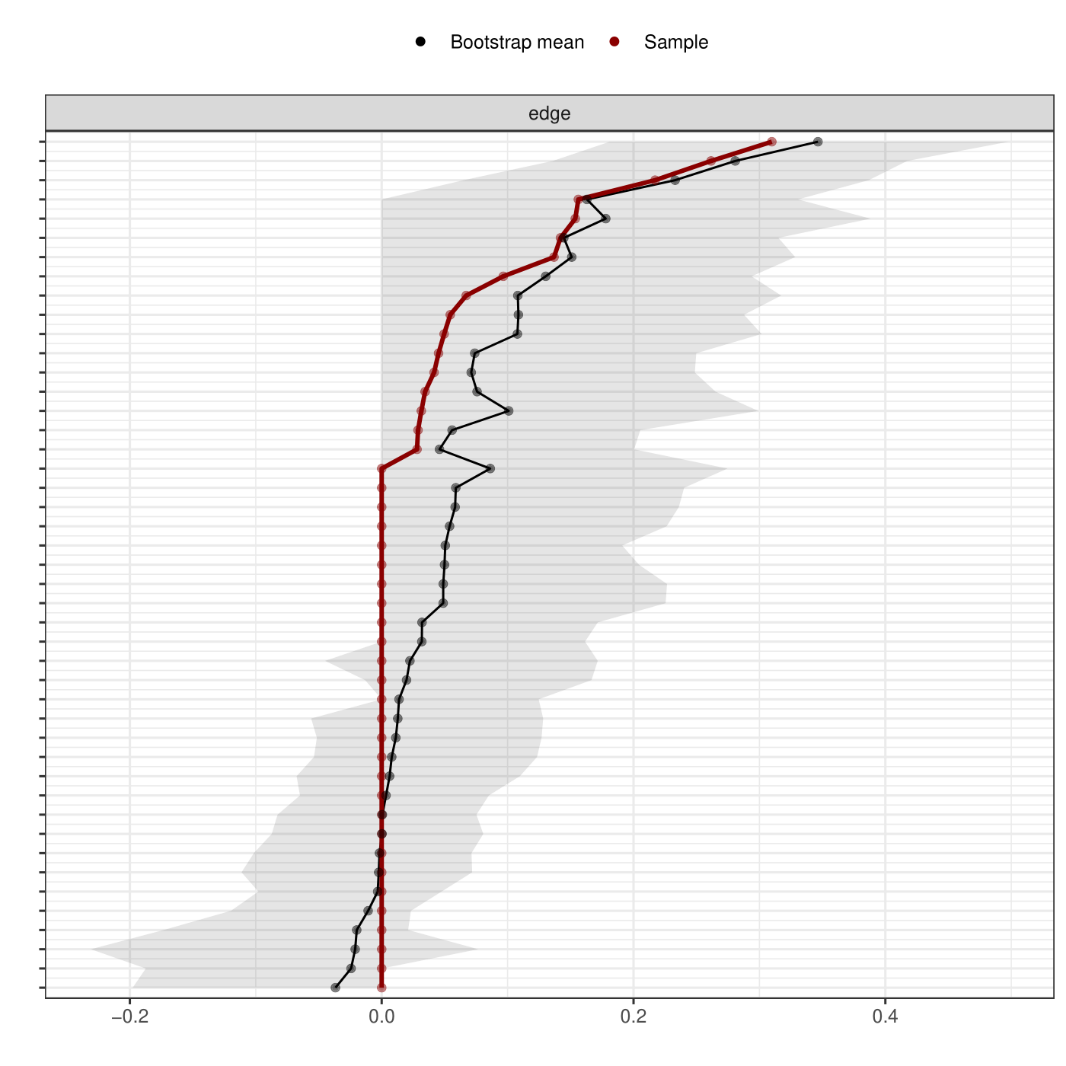
*Figure S2

*Edge Accuracy Analyses*

*Note.* The grey regions indicate the 95% confidence intervals and the red line represents edge values.

Figure S3

*Edge weights difference test*

*
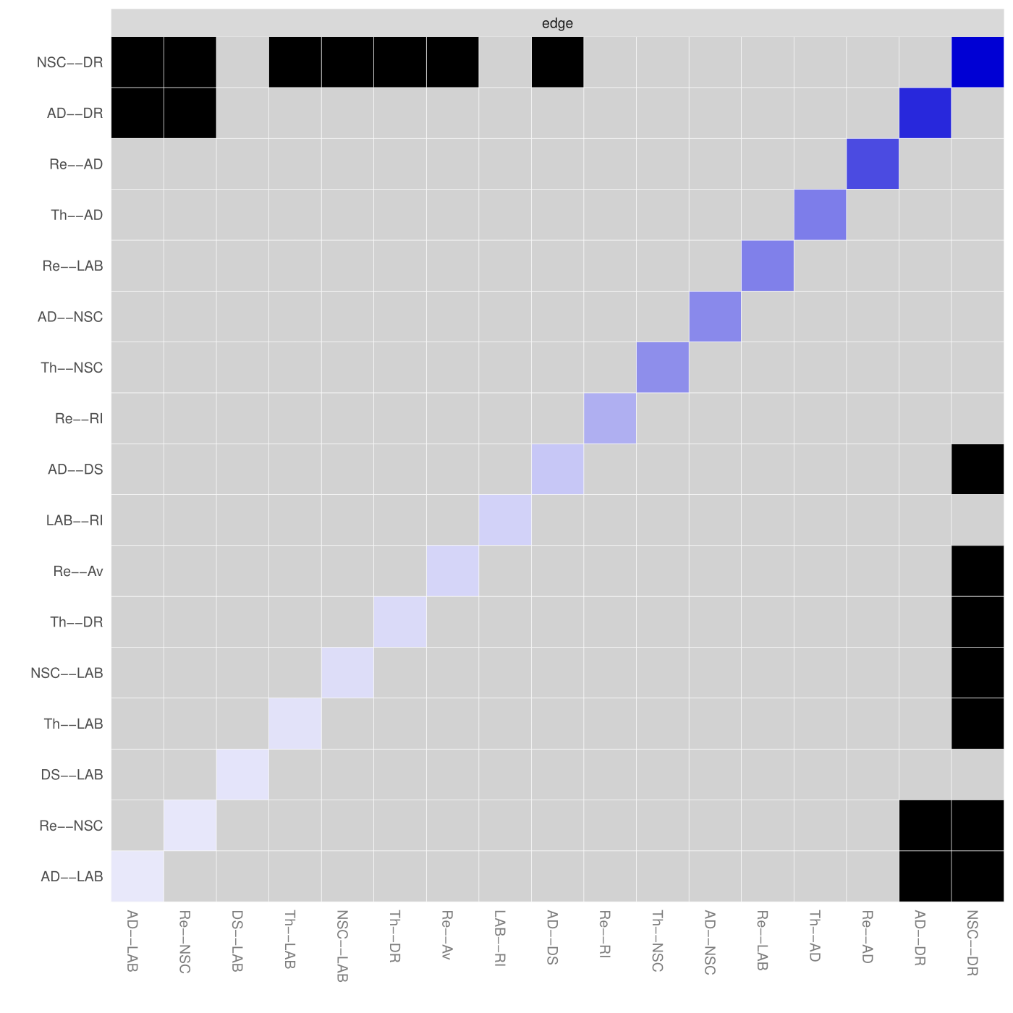
*

*Note.* Black boxes represent significant differences between edge weights.

*
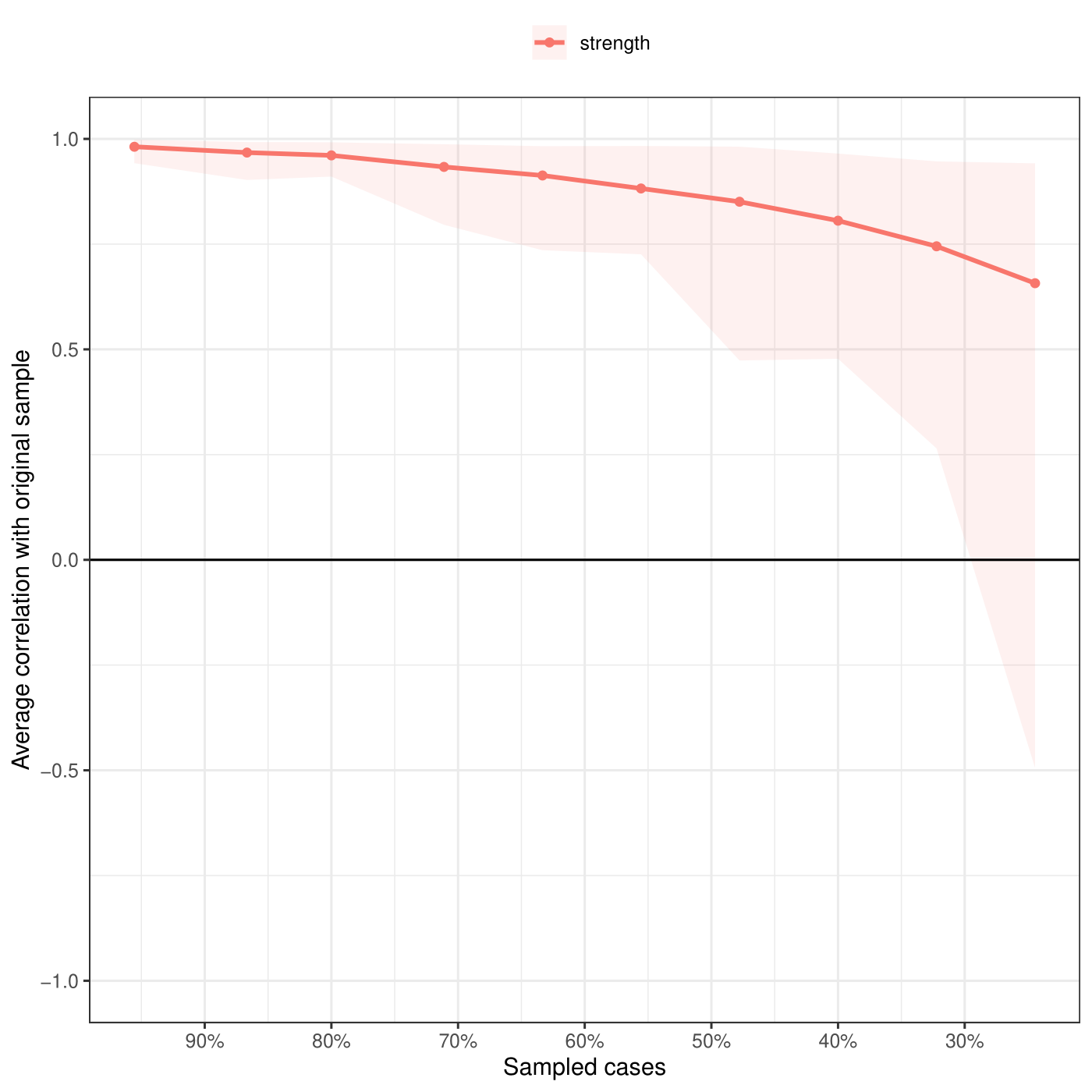
*Figure S4

*Centrality Stability Analysis*

*Note.* Average correlations between the original and centrality indices of network sampled with persons dropped sample. The red line indicate the means and areas indicate the range from the 2.5^th^ to the 97.5^th^ quantile.

Figure S5

*Centrality difference test*

*
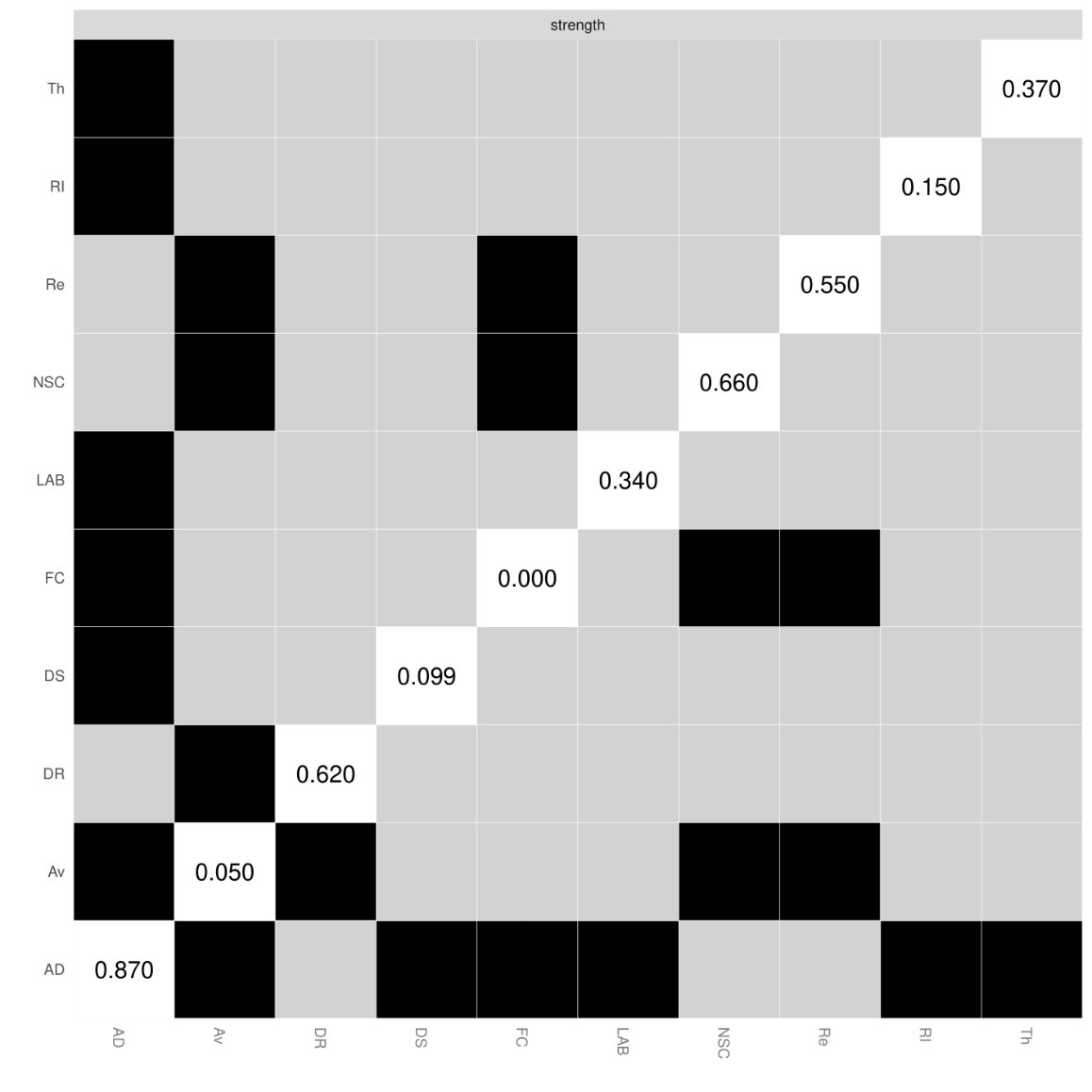
*

*Note.* The diagonal show standardized strength centrality values, black boxes represent significant differences in centrality estimates.

Table S4

*Codebook*

| **Position** | **Variable Name** | **Variable Label** | **Value** | **Value Label** | **Value indicating missing data** | **Question** |
| --- | --- | --- | --- | --- | --- | --- |
| 1 | TN | Participant | 1-102 | ID number | -99 | - |
| 2 | PMLD01_t0 | Communication difficulties | 1  2  3  4  5 | not at all  a little bit  moderatly  strongly  very strongly | -99 | In the last month I have experienced in Austria....  How much was the problem below true for you? |
| 3 | PMLD02_t0 | Discrimination |  |  |  |  |
| 4 | PMLD03_t0 | Conflicts with own or other ethnic groups in Austria |  |  |  |  |
| 5 | PMLD04_t0 | Family separation |  |  |  |  |
| 6 | PMLD05_t0 | Concern for family members remaining in the home country or living far away |  |  |  |  |
| 7 | PMLD06_t0 | Impossibility to travel home in case of emergency |  |  |  |  |
| 8 | PMLD07_t0 | Difficulties with work (e.g.: work permit, working conditions) |  |  |  |  |
| 9 | PMLD08_t0 | Difficulties with official channels (e.g.: interview with asylum agency) |  |  |  |  |
| 10 | PMLD09_t0 | Conflicts with authorities |  |  |  |  |
| 11 | PMLD10_t0 | No recognition as a refugee |  |  |  |  |
| 12 | PMLD11_t0 | Fear of future deportation to the homeland |  |  |  |  |
| 13 | PMLD12_t0 | Worries about not receiving medical support or treatment for health problems |  |  |  |  |
| 14 | PMLD13_t0 | Not having enough money to buy food, necessary clothing, or pay rent |  |  |  |  |
| 15 | PMLD14_t0 | Difficulties in obtaining financial support |  |  |  |  |
| 16 | PMLD15_t0 | Loneliness, boredom or isolation |  |  |  |  |
| 17 | PMLD16_t0 | Difficulties learning German |  |  |  |  |
| 18 | PMLD17_t0 | Difficulties in obtaining adequate housing |  |  |  |  |
| 19 | PMLD18_t0 | Family pressure, expectations which cannot be fulfilled ^a^ |  |  |  |  |
| 20 | PMLD19_t0 | No contact with family and friends in the country of origin ^a^ |  |  |  |  |
| 21 | PMLD20_t0 | Stressful media reports and social media content ^a^ |  |  |  |  |
| 22 | PMLD21_t0 | Negative media reports about Afghan fellow citizens in Austria ^a^ |  |  |  |  |
| 23 | PMLD22_t0 | Dependence on others due to language (loss of autonomy) ^a^ |  |  |  |  |
| 24 | PMLD23_t0 | Ethnic stigmatization ^a^ |  |  |  |  |
| 25 | PMLD24_t0 | Difficulties in understanding bureaucratic processes in Austria ^a^ |  |  |  |  |
| 26 | PMLD25_t0 | Different social norms than in the country of origin ^a^ |  |  |  |  |
| 27 | PMLD26_t0 | Homesickness ^a^ |  |  |  |  |
| 28 | ITQP01_t0 | Upsetting dreams | 0  1  2  3  4 | not at all  a little bit  moderately  quite a bit  extremely | -99 | P1. Having upsetting dreams that replay part of the experience or are clearly related to the experience? |
| 29 | ITQP02_t0 | Flashbacks |  |  |  | P2. Having powerful images or memories that sometimes come into your mind in which you feel the experience is happening again in the here and now? |
| 30 | ITQP03_t0 | Internal-Avoidance |  |  |  | P3. Avoiding internal reminders of the experience (for example, thoughts, feelings, or physical sensations)? |
| 31 | ITQP04_t0 | External-Avoidance |  |  |  | P4. Avoiding external reminders of the experience (for example, people, places, conversations, objects, activities, or situations)? |
| 32 | ITQP05_t0 | Being on guard |  |  |  | P5. Being “super-alert”, watchful, or on guard? |
| 33 | ITQP06_t0 | Startled |  |  |  | P6. Feeling jumpy or easily startled? |
| 34 | ITQP07_t0 | Functional impairment in relationships |  |  |  | P7. Affected your relationships or social life? |
| 35 | ITQP08_t0 | Functional impairment at work |  |  |  | P8. Affected your work or ability to work? |
| 36 | ITQP09_t0 | Functional impairment in other parts of life |  |  |  | P9. Affected any other important part of your life such as parenting, or school or college work, or other important activities? |
| 37 | ITQC01_t0 | Long time to calm down |  |  |  | C1. When I am upset, it takes me a long time to calm down. |
| 38 | ITQC02_t0 | Numb |  |  |  | C2. I feel numb or emotionally shut down. |
| 39 | ITQC03_t0 | Failure |  |  |  | C3. I feel like a failure. |
| 40 | ITQC04_t0 | Worthless |  |  |  | C4. I feel worthless. |
| 41 | ITQC05_t0 | Cut-off from others |  |  |  | C5. I feel distant or cut off from people. |
| 42 | ITQC06_t0 | Difficult to stay close to others |  |  |  | C6. I find it hard to stay emotionally close to people. |
| 43 | ITQC07_t0 | Functional impairment in relationships |  |  |  | C7. Created concern or distress about your relationships or social life? |
| 44 | ITQC08_t0 | Functional impairment at work |  |  |  | C8. Affected your work or ability to work? |
| 45 | ITQC09_t0 | Functional impairment in other parts of life |  |  |  | C9. Affected any other important parts of your life such as parenting, or school or college work, or other important activities? |

Table S5

R Code

| ##############################################################################  # #  # The boundaries between complex posttraumatic stress disorder symptom clusters and #  # post-migration living difficulties in traumatised Afghan refugees: a network analysis #  # #  # Jennifer Schiess-Jokanovic, Matthias Knefel, Viktoria Kantor, Dina Weindl, #  # Ingo Schäfer, and Brigitte Lueger-Schuster #  # submitted to Conflict & Health #  # #  ##############################################################################  #............................................................................................................................................................  **# Table of Contents**  #............................................................................................................................................................  # 1. Load libraries  # 2. Data preparation  # 3. Descriptive statistics  # 4. Network estimation  # 5. Stability and accuracy  # 6. Centrality estimates  #............................................................................................................................................................  **# 1. Load libraries**  #............................................................................................................................................................  library(“haven”)  library(“misty”)  library("openxlsx")  library("foreign")  library("psych")  library("ggplot2")  library("bootnet")  library("qgraph")  library(“mice”)  library("mgm")  library("networktools")  #............................................................................................................................................................  **# 2. Data preparation**  #............................................................................................................................................................  **## Load Data**  **## Check total file**  dim(dat)  names(dat)  head(dat)  tail(dat)  descript(dat)  names(dat)  na.descript(dat)  dat[dat==-99] <- NA  percentmissing = function(x){sum(is.na(x))/length(x) * 100}  missing = apply(dat, 1, percentmissing)  table(missing)  **## Assign and name variables to the questionnaire**  **### PMLDC**  PMLD_t0 <- cbind(dat$PMLD01_t0, dat$PMLD02_t0, dat$PMLD03_t0, dat$PMLD04_t0, dat$PMLD05_t0, dat$PMLD06_t0, dat$PMLD07_t0, dat$PMLD08_t0, dat$PMLD09_t0, dat$PMLD10_t0, dat$PMLD11_t0, dat$PMLD12_t0, dat$PMLD13_t0, dat$PMLD14_t0, dat$PMLD15_t0, dat$PMLD16_t0, dat$PMLD17_t0, dat$PMLD18_t0, dat$PMLD19_t0, dat$PMLD20_t0, dat$PMLD21_t0, dat$PMLD21_t0, dat$PMLD23_t0, dat$PMLD24_t0, dat$PMLD25_t0, dat$PMLD26_t0)  colnames(PMLD_t0) <- c("PMLD01_t0", "PMLD02_t0", "PMLD03_t0", "PMLD04_t0", "PMLD05_t0", "PMLD06_t0", "PMLD07_t0", "PMLD08_t0", "PMLD09_t0", "PMLD10_t0", "PMLD11_t0", "PMLD12_t0", "PMLD13_t0", "PMLD14_t0", "PMLD15_t0", "PMLD16_t0", "PMLD17_t0", "PMLD18_t0", "PMLD19_t0", "PMLD20_t0", "PMLD21_t0", "PMLD22_t0", "PMLD23_t0", "PMLD24_t0", "PMLD25_t0", "PMLD26_t0")  **### ITQ**  ITQ_t0 <- cbind(dat$ITQP01_t0, dat$ITQP02_t0, dat$ITQP03_t0, dat$ITQP04_t0, dat$ITQP05_t0, dat$ITQP06_t0, dat$ITQC01_t0, dat$ITQC02_t0, dat$ITQC03_t0, dat$ITQC04_t0, dat$ITQC05_t0, dat$ITQC06_t0)  colnames(ITQ_t0) <- c("Re1","Re2", "Av1", "Av2", "Th1", "Th2", "AD1", "AD2", "Nsc1", "Nsc2", "Dr1", "Dr2")  **## Handle missing values**  descript(PMLD_t0)  PMLD_imp <- mice(PMLD_t0, method = "pmm")  PMLD_mice <- complete(PMLD_imp)  descript(PMLD_mice)  PMLD_mice  ITQ_imp <- mice(ITQ_t0, method = "pmm")  ITQ_mice <- complete(ITQ_imp)  descript(ITQ_mice)  ITQ_mice  **## Assign to CPTSD Symptom Cluster & PMLD Factor**  **## CPTSD Symptom Cluster**  **###Re-experiencing**  Re_sum <- ITQ_mice$Re1 + ITQ_mice$Re2  **### Avoidance**  Av_sum <- ITQ_mice$Av1 + ITQ_mice$Av2  **### Sense of current threat**  Th_sum <- ITQ_mice$Th1 + ITQ_mice$Th2  **### Affective Dysregulation**  AD_sum <- ITQ_mice$AD1 + ITQ_mice$AD2  **### Negative Self-Concept**  Nsc_sum <- ITQ_mice$Nsc1 + ITQ_mice$Nsc2  **### Disturbances in relationships**  Dr_sum <- ITQ_mice$Dr1 + ITQ_mice$Dr2  **## PMLD Factors**  **### Discrimination & social-economical living conditions**  fac1_PMLD <- cbind(PMLD_mice$PMLD02_t0, PMLD_mice$PMLD13_t0, PMLD_mice$PMLD14_t0,PMLD_mice$PMLD17_t0)  **### Language acquisition & barriers**  fac2_PMLD <- cbind(PMLD_mice$PMLD01_t0, PMLD_mice$PMLD16_t0, PMLD_mice$PMLD19_t0, PMLD_mice$PMLD22_t0, PMLD_mice$PMLD24_t0)  **### Family concerns**  fac3_PMLD <- cbind(PMLD_mice$PMLD05_t0, PMLD_mice$PMLD26_t0, PMLD_mice$PMLD04_t0, PMLD_mice$PMLD06_t0)  **### Residence Insecurity**  fac4_PMLD <- cbind(PMLD_mice$PMLD10_t0, PMLD_mice$PMLD11_t0, PMLD_mice$PMLD21_t0)  fac1_PMLD_sum <-rowSums(fac1_PMLD, na.rm = TRUE)  fac2_PMLD_sum <-rowSums(fac2_PMLD, na.rm = TRUE)  fac3_PMLD_sum <-rowSums(fac3_PMLD, na.rm = TRUE)  fac4_PMLD_sum <-rowSums(fac4_PMLD, na.rm = TRUE)  #............................................................................................................................................................  **# 3. Descriptive statistics**  #............................................................................................................................................................  **## CPTSD Cluster**  descript(Re_sum)  descript(Av_sum)  descript(Th_sum)  descript(AD_sum)  descript(NSC_sum)  descript(DR_sum)  **## PMLD Factors**  descript(fac1_PMLD_sum)  descript(fac2_PMLD_sum)  descript(fac3_PMLD_sum)  descript(fac4_PMLD_sum)  #............................................................................................................................................................  **# 4. Network estimation**  #............................................................................................................................................................  **## Preparation**  Data_Study2_model3 <- cbind(Re_sum, Av_sum, Th_sum, AD_sum, Nsc_sum, Dr_sum, fac1_PMLD_sum, fac2_PMLD_sum, fac3_PMLD_sum, fac4_PMLD_sum)  as.data.frame(Data_Study2_model3)  colnames(Data_Study2_model3)<- c("Re", "Av", "Th", "AD", "NSC", "DR", "DS", "LAB", "FC", "RI")  colnames(Data_Study2_model3)  as.data.frame(Data_Study2_model3)  Names <- c("Re-experiencing", "Avoidance", "Sense of current threat", "Affective Dysregulation", "Negative Self-Concept", "Disturbances in Relationships", "Discrimination & Socio-econom.", "Language Acquisition & Barriers", "Family Concerns", "Residence Insecurity")  **## Correlation Matrix**  study2_model3.cor <- cor_auto(Data_Study2_model3)  study2_model3.cor  graph_model3.g <- qgraph(study2_model3.cor, label=colnames, graph="EBICglasso", layout="spring", cut=0, sampleSize= nrow(Data_Study2_model3), tuning=0, details=TRUE)  **## Network estimation**  network_model3 <- estimateNetwork(Data_Study2_model3, default = "EBICglasso", tuning=0, corMethod = c("cor_auto"))  plot(network_model3, layout = "spring", cut = 0, filename = "network_model1")  network_model3  **##Network visualization**  Network_plot1 <- qgraph(study2_model3.cor, graph = "EBICglasso", layout = "spring", tuning = 0, sampleSize = nrow(Data_Study2_model3),legend.cex = 0.4, vsize = 10, esize = 15, palette = "rainbow", posCol = "grey20", color = "lightsteelblue", negCol = "orange", borders = TRUE, border.color = "grey20", border.width = 1.5, vTrans = 250, details = FALSE, label.cex = 0.9, nodeNames = Names, filename = "Model_withoutgrouping", filetype = "pdf")  #............................................................................................................................................................  **# 5. Stability and accuracy**  #............................................................................................................................................................  **## Robustness analysis**  Boot1_model3 <- bootnet(network_model3, nBoots = 1000, nCores = 4) # ncores 4 oder 8?  plot(Boot1_model3, labels = FALSE, order = "sample")  plot(Boot1_model3, statistics = "edge", plot = "difference", onlyNonZero = TRUE, order = "sample")  save(Boot1_model3, file = "boot1_model1.Rdata")  summary(Boot1_model3)  **## Plot edge weight CI**  pdf("FigS1.pdf")  par(mfrow=c(2,2))  plot(Boot1_model3, labels = FALSE, order="sample")  par(mfrow=c(1,1))  dev.off()  **## Plot edge weight difference test**  pdf("FigS2.pdf")  par(mfrow=c(2,2))  plot(Boot1_model3, statistics = "edge", plot = "difference", onlyNonZero = TRUE, order = "sample")  par(mfrow=c(1,1))  dev.off()  Boot2_model3 <- bootnet(network_model3, nBoots = 1000, nCores = 5, type = "case")  plot(Boot2_model3)  save(Boot2_model3, file = "boot2_model3.Rdata")  corStability(Boot2_model3)  summary(Boot2_model3)  **# Plot Centrality stability**  pdf("FigS3.pdf")  par(mfrow=c(2,2))  plot(Boot2_model3)  par(mfrow=c(1,1))  dev.off()  **# Significant differences**  differenceTest(Boot1_model3, 1, 2,"strength") # Note:sign. diff. only between 3,4;  plot(Boot1_model3, "edge", plot = "difference", onlyNonZero = TRUE, order = "sample")  plot(Boot1_model3, "strength")  **#Plot sign. diff test**  pdf("FigS4.pdf")  par(mfrow=c(2,2))  plot(Boot1_model3, "edge", plot = "difference", onlyNonZero = TRUE, order = "sample")  par(mfrow=c(1,1))  dev.off()  pdf("FigS5.pdf")  par(mfrow=c(2,2))  plot(Boot1_model3, "strength")  par(mfrow=c(1,1))  dev.off()  #............................................................................................................................................................  **# 6. Centrality estimates**  #............................................................................................................................................................  centrality_auto(graph_model3.g)  centralityPlot(graph_model3.g, include = c("Strength", "Closeness", "Betweenness"))  bridge_model3 <- bridge(graph_model3.g, communities = c("1", "1", "1", "1", "1", "1","2", "2", "2", "2"))  bridge_model3  plot(bridge_model3)  caseDroppingBoot <- bootnet(network_model3,boots=1000,type="case", statistics="all", communities = c("1", "1", "1", "1", "1", "1","2", "2", "2", "2"))  summary(caseDroppingBoot)  nonParametricBoot <- bootnet(network_model3,boots=1000,type="nonparametric", statistics="all", communities = c("1", "1", "1", "1", "1", "1","2", "2", "2", "2"))  summary(nonParametricBoot)  corStability(caseDroppingBoot)  plot(caseDroppingBoot, statistics="bridgeExpectedInfluence")  plot(nonParametricBoot, statistics="bridgeExpectedInfluence")  plot(nonParametricBoot, statistics="bridgeExpectedInfluence", plot="difference")  summary_bridgeExpectedInfl <- summary(nonParametricBoot, statistics="bridgeExpectedInfluence")  save(summary_bridgeExpectedInfl, file = "pdf")  summary_bridgestrenght <- summary(nonParametricBoot, statistics = "bridgeStrength")  save(summary_bridgestrenght, file = "pdf") |
| --- |
